# Reviewer acknowledgement 2014

**DOI:** 10.1186/s13019-015-0227-8

**Published:** 2015-02-25

**Authors:** David Taggart, Vipin Zamvar

**Affiliations:** 1 John Radcliffe Hospital, Oxford, UK; 2 Royal Infirmary of Edinburgh, Edinburgh, UK

## Abstract

The Editors of the *Journal Cardiothoracic Surgery* would like to thank all of our reviewers who have contributed to the journal in Volume 10 (2014) and whose valuable support is fundamental to its success.

**Yasir Abu-Omar**

United Kingdom

**Gurkan Acar**

Turkey

**Deepak Acharya**

United States of America

**Werner Adler**

Germany

**George Aislaitner**

Greece

**Raja Akhtar**

Pakistan

**Mehmet Aktas**

United States of America

**Mohamed Alattar**

Egypt

**Keith Allen**

United States of America

**Rui Almeida**

Brazil

**Reda Al-Refaie**

Egypt

**Peter Alston**

United Kingdom

**Renato Alves**

Brazil

**Khalid Amer**

United Kingdom

**Kayvan Amjadi**

Canada

**Gianni Angelini**

United Kingdom

**Ashraf Anwar**

Saudi Arabia

**Richard Asinger**

United States of America

**Cansel Atinkaya Öztürk**

Turkey

**Rony Atoui**

Canada

**Alexandre Azmoun**

France

**Ugur Bal**

Turkey

**Nikolaos Baltayiannis**

Greece

**Gerardus Bennink**

Germany

**Marius Berman**

United Kingdom

**Jose Bernal**

Spain

**Jean-Philippe Berthet**

France

**Farah Bhatti**

United Kingdom

**Yavuz Bilgin**

Netherlands

**Adrian Billeter**

Germany

**Bojan Biocina**

Croatia

**Yasuyuki Bito**

Japan

**Nikolaos Bonaros**

Austria

**Johannes Bonatti**

United Arab Emirates

**Petre Vlah Horea Botianu**

Romania

**Christoph Brochhausen**

Germany

**Keith Buchan**

Uruguay

**Giovanni Cagnoni**

Italy

**Antonio Calafiore**

Saudi Arabia

**Luigi Camporota**

United Kingdom

**Luiz Fernando Caneo**

Brazil

**Khang Cao**

Viet Nam

**Giuseppe Cardillo**

Italy

**Thierry Carrel**

Switzerland

**Mario Castillo-Sang**

United States of America

**Betul Celik**

Turkey

**Rikhia Chakraborty**

United States of America

**Umar Chaudhry**

United Kingdom

**Arindam Chaudhury**

United States of America

**Jingjing Che**

China

**Haiquan Chen**

China

**Davy Cheng**

Canada

**Jian-Bo Cheng**

Taiwan

**Stephen Cheng**

Hong Kong

**Seong-Joon Cho**

Korea, South

**Se Hoon Choi**

Korea, South

**Jill Cholette**

United States of America

**Chee Rui Chong**

Brunei

**Torsten Christ**

Germany

**Namsik Chung**

Korea, South

**Sertac Cicek**

Turkey

**Oved Cohen**

Israel

**Steven Conrad**

United States of America

**Gioachino Coppi**

Italy

**Ricardo Corso**

Brazil

**Francisco Costa**

Brazil

**Carlos E Costa Almeida**

Portugal

**Michael Crittenden**

United States of America

**Shitao Cui**

China

**Martin Czerny**

Switzerland

**Yongch Daiyn**

China

**David D'Alessandro**

United States of America

**Petrov Danail**

Bulgaria

**Ahmad Darwazah**

Israel

**Seyed Hashem Davarpanah**

Iran

**Marisa De Feo**

Italy

**Thomas de Kroon**

Netherlands

**Indu Deglurkar**

United Kingdom

**Roland Demaria**

France

**Sanjay Deshmukh**

India

**Rogier Determann**

Netherlands

**Pascal Dohmen**

Germany

**Nicolas Doll**

Germany

**Kim Dong Kwan**

Korea, South

**Christophe Dooms**

Belgium

**Jakob Dörler**

Austria

**Jerry Easo**

Germany

**Holger Eggebrecht**

Germany

**Hussein EL Shafei**

United Kingdom

**John Elefteriades**

United States of America

**Adam EL-Gamel**

United Kingdom

**Shusuke Endo**

Japan

**Hideki Endoh**

Japan

**Joachim Mathias Erb**

Germany

**Maja Ercegovac**

Serbia

**Nevzat Erdil**

Turkey

**Neta Erez**

Israel

**Giampiero Esposito**

Italy

**Paulo Roberto Evora**

Brazil

**Dianchun Fang**

China

**Wentao Fang**

China

**Olivia Fanucchi**

Italy

**Khalil Fattouch**

Italy

**Michael Firstenberg**

United States of America

**Christophoros Foroulis**

Greece

**Omar Franco**

United States of America

**Robert Frater**

United States of America

**Richard K Freeman**

United States of America

**Sara Fuentes**

Spain

**Haruka Fujinami**

Japan

**Franco Gaboardi**

Italy

**Sean Galvin**

Australia

**Changqing Gao**

China

**Philippe Garot**

France

**Mario Gaudino**

Italy

**Walter Gomes**

Brazil

**Javier Gonzalez**

Spain

**Michael Goretsky**

United States of America

**Masashi Gotoh**

Japan

**Mustafa Guden**

Turkey

**Fernando Guimaraes**

Brazil

**Adem Guler**

Turkey

**Ahmet Umit Gullu**

Turkey

**Yanli Guo**

China

**Andreas Hagendorff**

Germany

**Michael Halkos**

United States of America

**Assad Haneya**

Germany

**Deborah Harrington**

United Kingdom

**Derek Hausenloy**

United Kingdom

**László Hejjel**

Hungary

**Khosro Hekmat**

Germany

**Roland Henaine**

France

**Anders Henriksson**

Sweden

**Mitsunori Higuchi**

Japan

**Dominique Himbert**

France

**Hitoshi Hirose**

United States of America

**Johannes Holfeld**

Austria

**Daijiro Hori**

Japan

**David Horne**

Canada

**Michael Hsin**

Hong Kong

**Nan-Yung Hsu**

Taiwan

**Han-Shui Hsu**

Taiwan

**Sheng-Shou Hu**

China

**Jinwook Hwang**

Korea, South

**Charles Ibingira**

Uganda

**Yasunori Iida**

Japan

**Kiyotaka Imoto**

Japan

**Andrea Imperatori**

Italy

**Tadashi Isomura**

Japan

**Hironori Izutani**

Japan

**Jan Janousek**

Czech Republic

**Robert Jaquiss**

United States of America

**Kalyana Javangula**

United Kingdom

**Mark Jones**

United Kingdom

**Maninder Kalkat**

United Kingdom

**Robin Kanagasabay**

United Kingdom

**Emmanouil Ioannis Kapetanakis**

Greece

**Mehmet Kaplan**

Turkey

**Oguz Karahan**

Turkey

**Theodoros Karaiskos**

Greece

**Tarkan Karakan**

Turkey

**Tara Karamlou**

United States of America

**Pankaj Kaul**

United Kingdom

**Toshihiro Kawahira**

Japan

**Steven Keller**

United States of America

**Alper Kepez**

Turkey

**John Kern**

United States of America

**Ken Kesler**

United States of America

**Dongyoon Keum**

Korea, South

**Zain Khalpey**

United States of America

**Arndt Kiessling**

Germany

**Yeongdae Kim**

Korea, South

**Hyun Koo Kim**

Korea, South

**Dae Hyun Kim**

Korea, South

**Nam Su Kim**

Korea, South

**Shuichi Kino**

Japan

**Alan Kirk**

United Kingdom

**Masahiro Kitada**

Japan

**Soichiro Kitamura**

Japan

**Robert Klautz**

Netherlands

**Chris Klonaris**

Greece

**Stefan Klotz**

Germany

**Junjiro Kobayashi**

Japan

**Toshiro Kobayashi**

Japan

**Philipp Kolat**

Germany

**Kemal Korkmaz**

Turkey

**Ioannis Koukis**

Greece

**Rafal Krenke**

Poland

**Rungroj Krittayaphong**

Thailand

**Jan Kuks**

Netherlands

**Giovanni Landoni**

Italy

**Rodney J Landreneau**

United States of America

**Frank Langer**

Germany

**John Lassetter**

United States of America

**Dong Kyu Lee**

Korea, South

**Sang-kwon Lee**

Korea, South

**Gunda Leschber**

Germany

**Thomas Lesser**

Germany

**Eric Lim**

United Kingdom

**Changyu Liu**

Chile

**Shangfeng Liu**

China

**Lunxu Liu**

China

**Filippo Lococo**

Italy

**Vladimir Lonsky**

Czech Republic

**Marco Lucchi**

Italy

**Heyman Luckraz**

United Kingdom

**Xiaofeng Lv**

China

**Andrius Macas**

Lithuania

**Ali Machaal**

United Kingdom

**Unnikrishnan Madathipat**

India

**Kayapanda Mandana**

India

**Peter Manning**

United States of America

**Brivaldo Markman-Filho**

Brazil

**Sven Martens**

Germany

**Luigi Martinelli**

Italy

**Ivo Martinovic**

Germany

**Antonio Martin-Ucar**

United Kingdom

**Giuseppe Marulli**

Italy

**Yoshiro Matsui**

Japan

**James McCully**

United States of America

**Gianclaudio Mecozzi**

Netherlands

**Lorenzo Menicanti**

Italy

**Praveen Menon**

India

**Kivanc Metin**

Turkey

**Antonio Miceli**

Italy

**Naoki Minato**

Japan

**Noritoshi Minematsu**

Japan

**Paul Modi**

United Kingdom

**Akira Mogi**

Japan

**Tamas F Molnar**

Hungary

**Marc Moon**

United States of America

**Roberto Mosca**

United Kingdom

**Ajay Moza**

Germany

**Boniface Msamati**

Tanzania

**Leonardo Mulinari**

Brazil

**Henrique Murad**

Brazil

**Ulf Myhre**

Malaysia

**Hiroshige Nakamura**

Japan

**Teruya Nakamura**

Japan

**Yoshitsugu Nakamura**

Japan

**Yuki Nakayama**

Japan

**Giovanni Nano**

Italy

**Ehsan Natour**

Netherlands

**Petr Nemec**

Czech Republic

**Vinicius Nina**

Brazil

**Luc Noyez**

Netherlands

**Jonah Odim**

United States of America

**Sunil Ohri**

United Kingdom

**Yutaka Okita**

Japan

**Jemi Olak**

United States of America

**Annette Olieman**

Netherlands

**Francesco Onorati**

Italy

**Korah Oommen**

United Kingdom

**Satoru Osaki**

United States of America

**Oner Ozdogan**

Turkey

**Hakan Ozkan**

Turkey

**Davide Pacini**

Italy

**Sanjay Pai**

India

**Nestoras Papadopoulos**

Germany

**Sotiris Papaspyros**

United Kingdom

**Kisung Park**

Korea, South

**Bernard Park**

United States of America

**Vanash Patel**

United Kingdom

**Marios Patronis**

United Kingdom

**Jason Peart**

Australia

**Sai Peasad**

United Kingdom

**Giles Peek**

United Kingdom

**Mate Petricevic**

Croatia

**Orlando Petrucci**

Brazil

**Rainer Petzina**

Germany

**Marcello Picchio**

Italy

**Maximilian Pichlmaier**

Germany

**Bruno Pinamonti**

Italy

**Bruno Podesser**

Austria

**Florentina Popescu**

United Kingdom

**Aron Frederik Popov**

Germany

**Tiziano Porretta**

Italy

**Italo Porto**

Italy

**Adriano Massimiliano Priola**

Italy

**Aristotelis Protopapas**

United Kingdom

**Rajesh Puranik**

Australia

**Yongjun Qian**

China

**Prem Rabindra**

United States of America

**Mohammad Taghi Rajabi Mashhadi**

Iran

**Mahesh Ramchandani**

United States of America

**Kandadai Seshadri Rammohan**

United Kingdom

**Giovanni Rapuzzi**

Italy

**Christoph Raspé**

Germany

**Darius Rassoulian**

Germany

**Alberto Redaelli**

Italy

**Attilio Renzulli**

Italy

**Oliver Reuthebuch**

Switzerland

**Eduardo Rocha**

Brazil

**Steffan Rodel**

Netherlands

**Sebastian Rojas**

Germany

**Alvaro Rojas-Peña**

United States of America

**Claudia Romagnoni**

Italy

**Arjang Ruhparwar**

Germany

**Elisabeth Rushing**

Switzerland

**Claudio Francesco Russo**

Italy

**Han Young Ryu**

Korea, South

**Eduardo Saadi**

Brazil

**Anton Sabashnikov**

Germany

**Rafael Sadaba**

Spain

**Bahram Sadeghi Bigham**

Iran

**Kamales Kumar Saha**

India

**Francesco Saia**

Italy

**Shun-Ichiro Sakamoto**

Japan

**Tomas Salerno**

United States of America

**Albert Sam**

United States of America

**Cleusa Santos**

Brazil

**Shigeki Sawada**

Japan

**Robert Scheubel**

Germany

**Ralph Schmid**

Switzerland

**Michael Rahbek Schmidt**

Denmark

**Stefan Schneider**

Germany

**Gilbert Schorlemmer**

United States of America

**Christian Schreiber**

Germany

**Christopher Seder**

United States of America

**Amir Sepehripour**

United Kingdom

**Paul Sergeant**

Belgium

**Aamir Shah**

United States of America

**Lisa Shah-Patel**

United States of America

**Hani Shennib**

United States of America

**Qiang Shi**

China

**Chih-chung Shiao**

Taiwan

**Chun Che Shih**

Thailand

**Satoshi Shiono**

Japan

**Saeed Shoar**

Iran

**Robert Siegel**

United States of America

**Shuli Silberman**

Israel

**Marcia Cristina Silva**

Brazil

**Scott Silvestry**

United States of America

**Pranava Sinha**

United States of America

**Leland Siwek**

United States of America

**Paul Soeding**

Australia

**Dimitrios Sokolis**

Greece

**Sebastian-Patrick Sommer**

Germany

**Joshua Sonett**

United States of America

**Gopal Soppa**

United Kingdom

**Sotirios Spiliopoulos**

Germany

**Francesco Squadrito**

Italy

**Umberto Squarcia**

Italy

**Elisabeth Stahle**

Sweden

**Joel Starkopf**

Estonia

**Pierluigi Stefano**

Italy

**Paul Stein**

United States of America

**Paul Stelzer**

United States of America

**R. Scott Stephens**

United States of America

**Sreekumar Subramanian**

United States of America

**Jie Sun**

China

**Jonas Sunden-Cullberg**

Sweden

**Yuji Tachimori**

Japan

**Shinzo Takamori**

Japan

**Masaya Tamura**

Japan

**Shinya Tane**

Japan

**Hong Tang**

China

**Giordano Tasca**

Italy

**Istemihan Tengiz**

Turkey

**Euclides Tenorio**

Brazil

**Michael Teodori**

United States of America

**Nils Teucher**

Germany

**Jess Thompson**

United States of America

**Sarah K Thompson**

Australia

**Tom Treasure**

United Kingdom

**Hiroyoshi Tsubochi**

Japan

**Tomohiro Tsunekawa**

Japan

**Yasuhiro Tsutani**

Japan

**Joseph Turek**

United States of America

**James Tweddell**

United States of America

**Alper Ucak**

Turkey

**Kazuhiro Ueda**

Japan

**Yuichi Ueda**

Japan

**Hidetaka Uramoto**

Japan

**Elijah Ussiri**

Tanzania

**Jarle Vaage**

Norway

**Gonzalo Varela**

Spain

**Dirk Varelmann**

United States of America

**Prem Venugopal**

United Kingdom

**Giulia Veronesi**

Italy

**Murali Vettath**

India

**Nicola Viola**

United Kingdom

**Emiliano Votta**

Italy

**Alexander Wahba**

Norway

**Matthew Wall**

United States of America

**Qing Wang**

China

**Rong Wang**

China

**Zhiquan Wang**

United States of America

**Atsushi Watanabe**

Japan

**Bo Wei**

China

**Douglas West**

United Kingdom

**Anne Westerlind**

Sweden

**Alexander Weymann**

Germany

**Elizabeth Whitlock**

United States of America

**Yu-Chung Wu**

Taiwan

**Harry Hua-Xiang Xia**

United States of America

**Cangsong Xiao**

China

**Fei Xing**

United States of America

**Quansheng Xingqs**

China

**Fikri Yapici**

Turkey

**Guilherme Yazbek**

Brazil

**Ali Umit Yener**

Turkey

**Malik Yilmaz**

Turkey

**Hiroyasu Yokomise**

Japan

**Chen Yongbing**

China

**Lei Yu**

China

**Afzal Zaidi**

United Kingdom

**Farsad Zamani Boroujeni**

Iran

**Hongqiang Zhang**

China

**Wayne Zhang**

United States of America

**Wolfgang Zink**

Germany

